# Laparoscopy-Assisted Transgastrointestinal Endoscopic Retrieval of a Denture Impacted in the Esophagus: A Case Report

**DOI:** 10.7759/cureus.55701

**Published:** 2024-03-07

**Authors:** Daichi Nitta, Shunji Kinuta

**Affiliations:** 1 Surgery, Takeda General Hospital, Aizuwakamatsu, JPN

**Keywords:** esophageal foreign body, esophageal impaction, denture ingestion, transgastrointestinal endoscopy, laparoscopy-assisted surgery

## Abstract

We report the management of a complex case involving a 66-year-old male with significant medical comorbidities who inadvertently swallowed a denture, leading to unsuccessful endoscopic removal attempts. CT scans revealed an esophageal perforation. Following an initial unsuccessful conventional endoscopic attempt, we employed a laparoscopy-assisted transgastrointestinal endoscopic approach. This novel technique facilitated the successful retrieval of the denture without further esophageal damage, underscoring its utility in managing challenging esophageal foreign bodies. The patient’s postoperative recovery was uneventful, highlighting the safety and effectiveness of the procedure.

## Introduction

Most esophageal foreign bodies can be easily removed by endoscopy [1]. However, artificial dentures with sharp clasps have been reported to cause esophageal mucosal impaction and, ultimately, perforation, with major consequences [2,3].

## Case presentation

**Figure 1:**
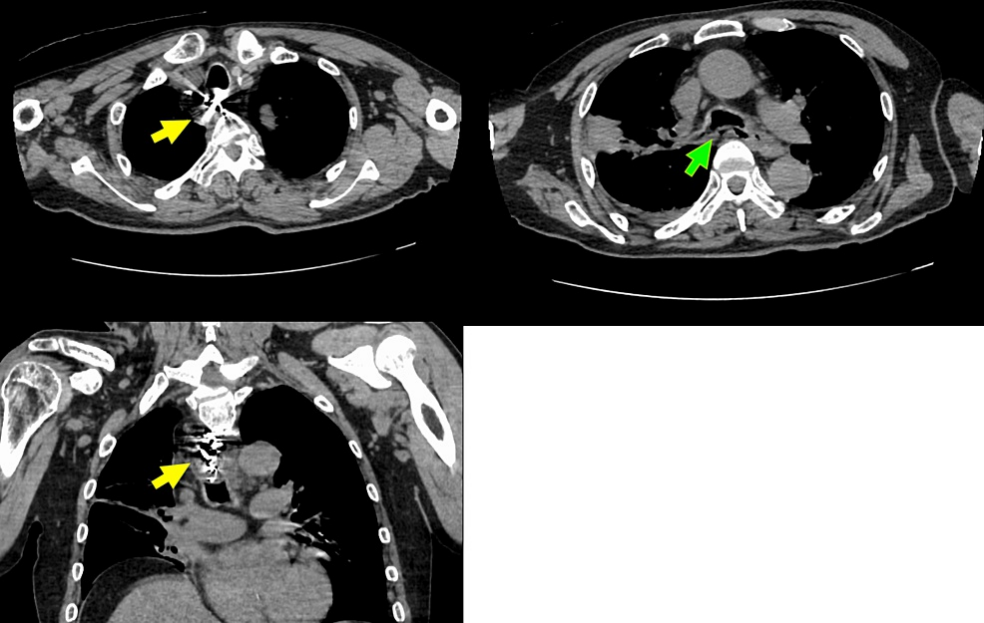
Chest CT findings: presence of a denture within the esophageal lumen and extraluminal air adjacent to the esophagus.

A 66-year-old male presented with a chief complaint of a foreign body in the esophagus. His past medical history included type 2 diabetes mellitus, cerebral infarction, symptomatic epilepsy, and intellectual disability, with no significant family history noted. On October 2, X year, the patient inadvertently ingested a partial denture during dinner, leading to referral to our hospital due to the complexity of endoscopic removal. The initial examination showed a height of 165.0 cm, weight of 53.3 kg, blood pressure of 138/86 mmHg, pulse of 101 beats/minute, and temperature of 37.6°C. The abdomen was flat, soft, and without tenderness, recoil pain, or muscular defense. Initial blood tests revealed an elevated inflammatory response with a white blood cell count of 17,500/µL and a C-reactive protein level of 20.45 mg/dL. A simple CT scan of the chest revealed a denture in the esophagus and air outside the esophagus (Figure 1), indicating the possibility of esophageal perforation due to the accidental ingestion of a hooked denture, which initiated treatment under general anesthesia with contemplation of surgical intervention.

**Figure 2:**
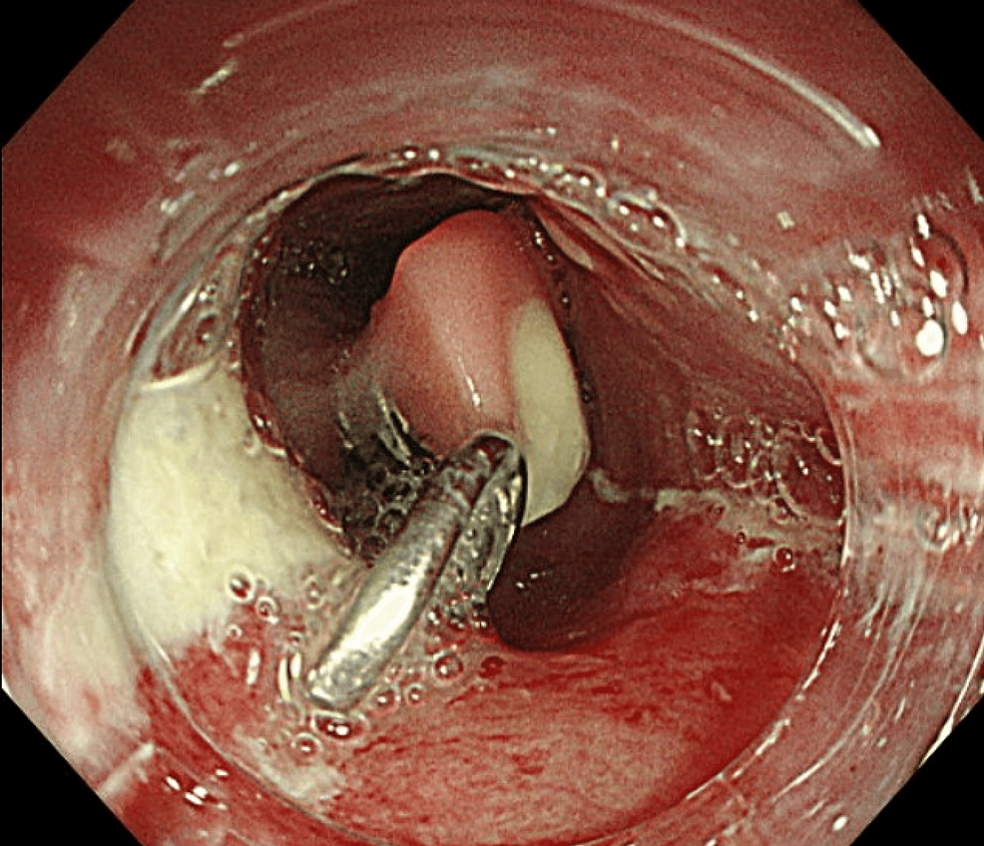
Esophageal presence of a barbed denture: a denture with hooks perforating the esophagus, with the hooks facing toward the oral direction and causing perforation on the distal side.

Bronchoscopic findings showed compression of the membranous part of the main bronchus from the esophageal side without evident perforation. Upper endoscopic findings revealed a hooked denture perforated the esophagus on the anorectal side (Figure 2), with extraction proving challenging despite attempts using forceps. Given the difficulty of oral removal, a transgastric endoscopic operation was deemed more feasible, guided by the shape of the fittings enabling removal from the anorectal side.

**Figure 3:**
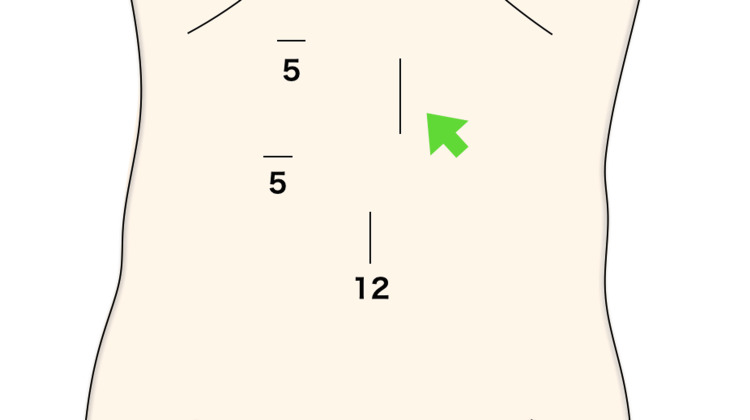
Three ports: 12 mm at the umbilicus, 5 mm at the right cardiac fossa, and 5 mm below the right costal arch. The green arrow indicates the endoscope insertion opening and enterostomy site.

Surgery commenced under general anesthesia with three ports: 12 mm at the umbilicus, 5 mm at the right cardiac fossa, and 5 mm below the right costal arch (Figure 3).

**Figure 4:**
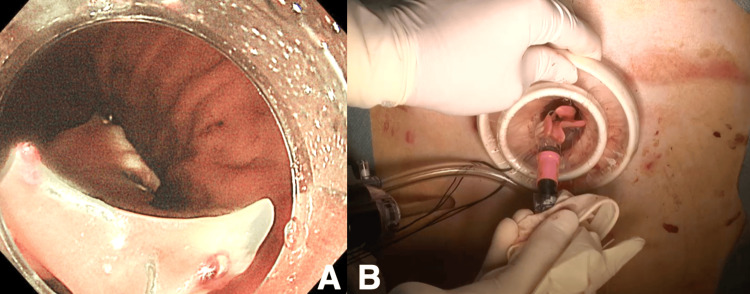
A: A hooked denture was extracted from the esophagus into the stomach. B: A hooked denture was subsequently removed from the stomach.

Laparoscopy confirmed the feasibility of elevating the lower part of the anterior wall of the gastric body to the abdominal wall. The procedure involved a skin incision directly above the determined site, followed by the insertion of an endoscope retrograde to the gastroesophageal junction. The hooked denture, perforating the mucosa, was successfully extracted (Figure 4), and the endoscopic operation concluded with suturing and closure. On postoperative day 12, CT showed improvement of mediastinitis, and the patient was transferred to the hospital due to good progress and bedridden condition.

## Discussion

Dentures lodged in the esophageal wall may cause serious complications, including fistula formation and perforation [1-3]. Reports indicate that the incidence of complications increases dramatically 24 hours post-ingestion, from 3.2% to a maximum of 23.5% after 48 hours [4]. The occurrence rate of strictures has been reported as 66.6% at the esophageal inlet, 19% at the tracheal bifurcation, and 14.3% at the esophagogastric junction [5]. Many patients in reported cases were elderly and had neurological disorders such as dementia, sequelae of cerebral hemorrhage, schizophrenia, cerebral infarction, or cerebral palsy [6]. Dentures without small fasteners typically pass safely through the esophagus, stomach, and intestines to the anus. However, dentures with sharp fasteners may damage the mucosa of the pharynx and esophagus [7-9]. Therefore, endoscopic removal of dentures is difficult, and, if left untreated, it can lead to esophageal compression necrosis and ultimately fatal complications such as esophageal perforation and mediastinitis.

This case involves an elderly male with intellectual disability and cerebral infarction, complicating the removal of a 5 cm long hooked denture via upper gastrointestinal endoscopy. Oral endoscopy remains the primary choice for minimally invasive treatment of esophageal foreign bodies, offering diagnostic and therapeutic benefits. The choice between rigid and flexible endoscopes depends on the location and characteristics of the foreign body. In cases of hooked dentures, especially larger ones, oral endoscopic extraction may prove challenging. This case, involving a 5 cm long hooked denture, encountered difficulties with forceps extraction during an attempted oral endoscopy. Despite subsequent attempts at endoscopic removal upon transfer to our hospital, resistance was encountered, prompting the adoption of a transgastric endoscopic approach. This alternative method enabled denture removal from the antrum without inducing esophageal perforation. Enterostomy, chosen for postoperative nutritional management, was preferred over gastrostomy in consideration of the observed air outside the esophagus on CT, indicating potential esophageal perforation. The decision aimed to avoid complications associated with oral nutrition in cases of suspected perforation. Intraoperative and postoperative assessments revealed no esophageal perforation, affirming the safety and relatively minimally invasive nature of the procedure.

A search on PubMed from 2010 to 2024 using the keywords “denture,” “endoscope,” and “oesophagus” yielded reports of cases involving excision via the cervical approach and extraction utilizing the Weerda diverticuloscope [10,11]. Similarly, a search in Japanese medical central journals from 2010 to 2024 using the keywords “denture,” “endoscope,” and “oesophagus” revealed reports of cases where extraction was conducted through midline upper abdominal incision with insertion of both oral and gastric endoscopes, as well as cases of extraction via gastrostomy endoscopic operation [12,13]. However, there were no similar case reports to our own experience of laparoscopy-assisted transgastric endoscopic removal of a challenging denture.

## Conclusions

The successful retrieval of an impacted denture through a laparoscopy-assisted transgastrointestinal endoscopic approach showcases an innovative and effective method for handling complex esophageal foreign bodies. This minimally invasive technique offers a significant advantage over traditional methods, particularly for cases where conventional endoscopy faces challenges due to the shape or size of the foreign object. Providing a safer alternative that reduces mucosal damage and esophageal perforation risks, this case enriches the gastrointestinal surgery field. It underscores the role of adaptability and interdisciplinary collaboration in enhancing patient outcomes.
